# Role of high-mobility group box 1 in methamphetamine-induced activation and migration of astrocytes

**DOI:** 10.1186/s12974-015-0374-9

**Published:** 2015-09-04

**Authors:** Yuan Zhang, Tiebing Zhu, Xiaotian Zhang, Jie Chao, Gang Hu, Honghong Yao

**Affiliations:** Department of Pharmacology, Medical School of Southeast University, Nanjing, 210009 China; Department of Cardiology, The First Affiliated Hospital of Nanjing Medical University, Nanjing, Jiangsu 210029 China; Department of Physiology, Medical School of Southeast University, Nanjing, China; Department of Pharmacology, Nanjing Medical University, Nanjing, China; Institute of Life Sciences, Key Laboratory of Developmental Genes and Human Disease, Southeast University, Nanjing, Jiangsu China

**Keywords:** HMGB1, Methamphetamine, C6 astroglia-like cells, Activation, Migration

## Abstract

**Background:**

Mounting evidence has indicated that high-mobility group box 1 (HMGB1) is involved in cell activation and migration. Our previous study demonstrated that methamphetamine mediates activation of astrocytes via sigma-1 receptor (σ-1R). However, the elements downstream of σ-1R in this process remain poorly understood. Thus, we examined the molecular mechanisms involved in astrocyte activation and migration induced by methamphetamine.

**Methods:**

The expression of HMGB1, σ-1R, and glial fibrillary acidic protein (GFAP) was examined by western blot and immunofluorescent staining. The phosphorylation of cell signaling pathways was detected by western blot, and cell migration was examined using a wound-healing assay in rat C6 astroglia-like cells transfected with lentivirus containing red fluorescent protein (LV-RFP) as well as in primary human astrocytes. The role of HMGB1 in astrocyte activation and migration was validated using a siRNA approach.

**Results:**

Exposure of C6 cells to methamphetamine increased the expression of HMGB1 via the activation of σ-1R, Src, ERK mitogen-activated protein kinase, and downstream NF-κB p65 pathways. Moreover, methamphetamine treatment resulted in increased cell activation and migration in C6 cells and primary human astrocytes. Knockdown of HMGB1 in astrocytes transfected with HMGB1 siRNA attenuated the increased cell activation and migration induced by methamphetamine, thereby implicating the role of HMGB1 in the activation and migration of C6 cells and primary human astrocytes.

**Conclusions:**

This study demonstrated that methamphetamine-mediated activation and migration of astrocytes involved HMGB1 up-regulation through an autocrine mechanism. Targeting HMGB1 could provide insights into the development of a potential therapeutic approach for alleviation of cell activation and migration of astrocytes induced by methamphetamine.

## Background

Despite the advances in intensive care and the development of pharmacological agents that inhibit methamphetamine-induced neurotoxicity, FDA-approved pharmacotherapies for treatment of negative effects of methamphetamine are still lacking. As an addictive pharmacological psychostimulant, methamphetamine is one of the most commonly abused agents by illicit drug users [1, 2]. In addition to its immediate stimulant effects, such as euphoria and enhanced energy, methamphetamine use also manifests clinical psychiatric symptoms characterized by cognitive deficits, depression, anxiety, psychotic symptoms, and motor deficits because of its neurotoxic effect [2]. Accumulated evidence suggests that there is a close relationship between methamphetamine-induced neurotoxicity and activated astrocytes. Previous studies from our group and others have indicated that astrocyte activation is involved in methamphetamine-mediated neurotoxicity [3, 4].

Astrocytes are the most abundant cell type within the central nervous system (CNS) and may play diverse roles in regulating and maintaining CNS homeostasis [5, 6]. In addition to their normal physiological functions, astrocytes can be pathologically activated, and they are characterized by abnormal morphology with reactive astrogliosis [7–11]. One of the major cellular manifestations of astrocyte inflammatory responses is reactive astrogliosis, in which astrocytes undergo rapid proliferation and enhanced migration toward the site of inflammation and attempt to mitigate collateral damage by isolating the damaged area [8, 12, 13]. Previous studies have demonstrated the presence of astrocyte activation in the striatum of methamphetamine-treated mice and rats in vivo [4, 14] as well as in in vitro systems [15, 16]. Methamphetamine is known to exhibit moderate affinity for sigma-1 receptor (σ-1R), which is expressed in most neuronal cells [17]. σ-1R is a unique drug-binding protein that is present in the CNS and in the periphery [18]. Our previous study demonstrated that methamphetamine-mediated activation of astrocytes involves the up-regulation of σ-1R through a positive feedback mechanism. However, the mechanisms underlying the downstream pathways remain poorly understood.

High-mobility group box 1 (HMGB1) is a non-histone DNA-binding protein that regulates gene expression and nucleosome stabilization [19]. HMGB1 is also a cytokine that can activate monocytes and neutrophils involved in inflammation. Currently, HMGB1 is thought to be a cytokine-like molecule when it is released from activated macrophages, dendritic cells, and natural killer cells [20, 21]. A previous study has reported that HMGB1 promotes the proliferation and migration of glioma cells [22]. In the CNS, HMGB1 serves as a danger signal that evokes inflammatory reactions by activation of various immune-related cells, including microglia [23]. Moreover, HMGB1 secreted from astrocytes promotes endothelial progenitor cell-mediated neurovascular remodeling and enhances the accumulation of endothelial progenitor cells during stroke recovery [24, 25]. Hayakawa et al. also reported that reactive astrocytes promote adhesive interactions between the brain endothelium and endothelial progenitor cells via HMGB1 and β-2 integrin signaling [26]. Despite extensive studies, it is unclear whether HMGB1 plays a critical role in methamphetamine-induced neurotoxicity. Based on these findings, we hypothesized that methamphetamine activates astrocytes through an autocrine mechanism(s) by up-regulating the expression of HMGB1.

Thus, the present study sought to determine whether HMGB1 is involved in the astrocyte activation and migration induced by methamphetamine. In the current study, we provide direct evidence that methamphetamine induces astrocyte activation and migration, thereby contributing to neuroinflammation in drug abusers via a previously unidentified autocrine pathway that leads to increased HMGB1 expression.

## Methods

### Cell culture

Rat C6 astroglia-like cells were obtained from the European Collection of Cell Cultures. C6 cells were grown in Dulbecco’s modified Eagle’s medium (DMEM) supplemented with 10 % heat-inactivated fetal bovine serum (FBS) and 1 % penicillin/streptomycin. Primary human astrocytes were purchased from ScienCell (Carlsbad, CA, USA) and cultured in the astrocyte medium (ScienCell). Cells were grown in a CO_2_ incubator (Thermocon Electron Corporation, Waltham, MA, USA) at 37 °C in an atmosphere of 95 % air and 5 % CO_2_ with 98 % humidity.

### Reagents

Methamphetamine was purchased from the National Institute for the Control of Pharmaceutical and Biological Products (Beijing, China). The specific Src kinase inhibitor (PP2), ERK1/2 inhibitor (U0126), and Ikk-2 inhibitor (SC514) were purchased from Calbiochem (San Diego, CA, USA). The concentrations of these inhibitors were based on the concentration-curve study and our previous reports [3].

### MTT assay

The MTT assay was performed to measure cell viability. Briefly, cells were seeded in 96-well plates, and MTT dye was added 1.5 h before the termination of experiment. Optical density (OD) was acquired at 570 nm by Synergy H1 Multi-Mode Reader (BioTek, Winooski, VT, USA).

### CCK8 assay

The cell viability was measured by Cell Counting Kit 8 (CCK8) from YEASEN (Shanghai, China). Cells were plated at a density of 2 × 10^4^ cells/well on 96-well plates. After exposure to meth for 24 h, CCK-8 (10 μl) was added to each well of 96-well plate and the plate was incubated for 1.5 h at 37 °C. Viable cells were counted by absorbance measurements at 450 nm using a Synergy H1 Multi-Mode Reader (BioTek, Winooski, VT, USA).

### Western blot

Total protein was isolated from C6 cells or primary human astrocytes using ice-cold RIPA buffer. Total protein concentrations were measured with the BCA Protein Assay Kit (Pierce, Rockford, IL, USA). Protein samples (30 μg per lane) were separated using SDS-PAGE and transferred to polyvinylidene difluoride (PVDF) membranes. Proteins were detected by incubation with primary antibodies (p-Src/Src, p-ERK/ERK, NF-κB p65, p-NF-κB p65, histone H3, or GAPDH at 1:1000 from Cell Signaling, Danvers, MA, USA; or σ-1R at 1:500 from Invitrogen, Carlsbad, USA) followed by secondary antibodies (horseradish peroxidase-conjugated to goat anti-mouse/rabbit IgG at 1:2,000). Glial fibrillary acidic protein (GFAP) and β-actin (1:1000; Sigma-Aldrich, St. Louis, MO, USA) were employed as loading controls. Immunoblots were visualized using Millipore ECL Western Blotting Detection System (Millipore, Billerica, MA, USA). Signals were detected by chemiluminescence and imaged on the Microchemi 4.2 (DNR, Israel) digital image scanner. Quantification was performed by densitometry using Image J software (NIH).

### siRNA experiment

Control siRNA, human σ-1R siRNA (sc-42250), human Src siRNA (sc-29228), human NF-κB p65 siRNA (sc-29410), and rat HMGB1 siRNA (sc-270015) were obtained from Santa Cruz Biotechnology (Dallas, TX, USA). Signal Silence® p44/42 MAPK (Erk1/2) siRNA was purchased from Cell Signaling (Danvers, MA, USA). The siRNAs were prepared according to the transfection protocol for cell cultures from Santa Cruz Biotechnology. Briefly, 1 ml of siRNA transfection reagent mixture (transfection reagent, sc-29528; transfection medium, sc-36868) was co-incubated with C6 cells for 5 h in a 5 % CO_2_ incubator at 37 °C, and an equal amount of DMEM with 20 % FBS was then added. An additional incubation was performed for 18 h, and the procedure for conditioned media was then performed.

### Immunofluorescence staining

Cells were cultured on cover-slips and then treated with methamphetamine for 12 h. Cells were fixed with 4 % paraformaldehyde and then permeabilized with 0.3 % Triton X-100 in phosphate-buffered saline (PBS). After the cells were blocked with 10 % normal goat serum (NGS) in 0.3 % Triton X-100, cells were incubated with mouse anti-GFAP antibodies (1:800; Sigma-Aldrich, St. Louis, MO, USA) overnight at 4 °C. Cells were then incubated with the AlexaFluor 488-conjugated anti-mouse IgG secondary antibody (1:250; Invitrogen, Carlsbad, USA). GFAP expression was observed using a fluorescence microscope (Zeiss, Carl Zeiss, Göttingen, Germany). The quantification of fluorescence intensity was performed using Image J software.

### Lentiviral transduction of C6 astrocytes

C6 cells were transduced with a lentivirus containing red fluorescent protein (LV-RFP) from Hanbio Inc. (Shanghai, China). The cells were trypsinized and washed with DMEM (no FBS) twice. The cells (1 × 10^5^) were then cultured with 8 μg/ml polybrene and 2 μl of LV-RFP solution (3 × 10^8^ IU/ml) in 500 μl of DMEM (10 % FBS) in each well of a 24-well plate. After incubation at 37 °C in 5 % CO_2_ for 24 h, the treatment medium was replaced with fresh DMEM containing 10 % FBS. Once confluence greater than 50 % was reached, the transduced cells were selected using 10 μg/ml puromycin. The cells were subsequently washed twice with fresh DMEM containing 10 % FBS. The pure, transduced cells were expanded and stored in liquid nitrogen as previously described [27].

### Cell migration assay

The cell migration capability was examined using a wound-healing assay. Cells were seeded in a 24-well plate and incubated to 70–80 % confluence. A cell-free straight line was then created in the center of the well by scratching with a sterile 200-μl pipette tip. Similarly, a second straight line was scratched perpendicular to the first line to create a cross-shaped cellular gap in each well. Cells were treated with methamphetamine and then allowed to migrate into the cell-free wound for 24 h. Digital images of the cell gap were captured at different time points, and the gap width was quantitatively evaluated using Image J software.

### Statistical analysis

Statistical analysis was performed using SigmaPlot software (SigmaPlot 11.0, Systat. Software Inc., San Jose, California, USA). Data were presented as the mean ± SD. Significance of differences between control and samples treated with various drugs was determined by one-way ANOVA, and Tukey’s post hoc test and Bonferroni correction were used for multiple comparisons. *P* values < 0.05 were considered as statistically significant.

## Results

### Methamphetamine mediates the expression of HMGB1 in astrocytes

**Fig. 1:**
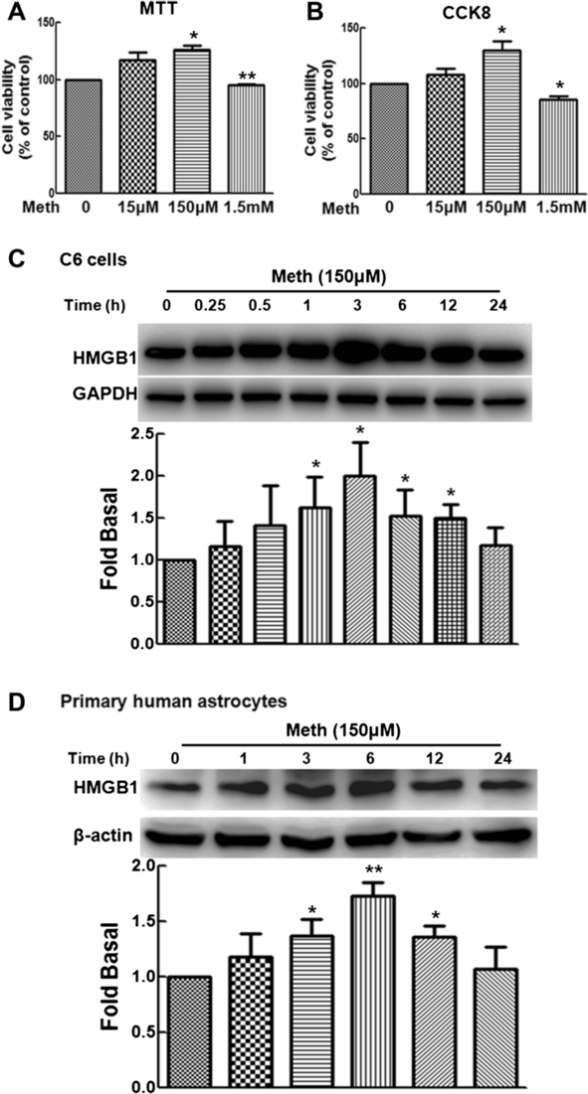
Methamphetamine induced the expression of HMGB1 in astrocytes. Methamphetamine increased the cell proliferation of C6 cells. Cells were exposed to different concentrations of methamphetamine (15 μM, 150 μM, and 1.5 mM) for 24 h followed by the MTT assay (**a**) and CCK8 (**b**). Methamphetamine-induced HMGB1 expression in a time-dependent manner in C6 cells (**c**) and primary human astrocytes (**d**). All the data are presented as the mean ± SD of three individual experiments. *p < 0.05 and **p < 0.01 compared with control group

Because reactive astrocytes undergo rapid proliferation [8, 12, 13], we first investigated the effect of methamphetamine on cell proliferation in C6 cells. Cells were exposed to different concentrations of methamphetamine (15 μM, 150 μM, and 1.5 mM) for 24 h followed by cell viability assessment. As shown in Fig. 1a, the cell proliferation of astrocytes was significantly increased with 150 μM methamphetamine, whereas cell viability decreased after treating with 1.5 mM methamphetamine. In addition to the MTT assay, the effect of methamphetamine on the cell viability of C6 cells was further corroborated by CCK8 cell proliferation assay. As shown in Fig. 1b, treatment of C6 cells with 150 μM methamphetamine significantly increased the viability by 135 %. To explore the potential target proteins involved in astrocytic proliferation, the expression of HMGB1 was detected by western blot analysis. As shown in Fig. 1c, methamphetamine treatment resulted in increased expression of HMGB1 with the peak response at 3 h. This finding was further confirmed in primary human astrocytes, methamphetamine also induced the expression of HMGB1 (Fig. 1d). Therefore, methamphetamine treatment increased cell proliferation and HMGB1 expression in astrocytes.

### Methamphetamine mediates the activation of the Src/ERK MAPK pathway

**Fig. 2:**
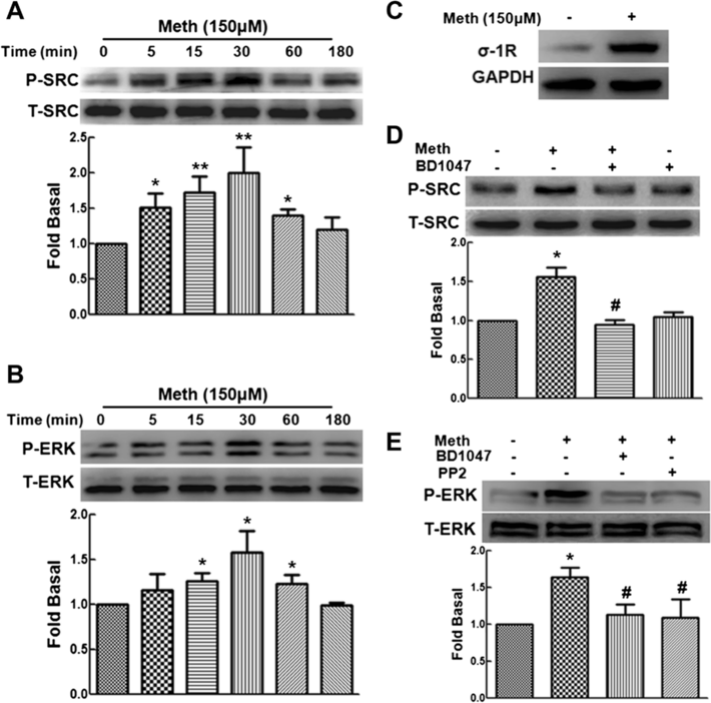
Methamphetamine mediates the activation of the Src/ERK MAPK pathway. Methamphetamine induced **a** Src phosphorylation and **b** ERK phosphorylation in a time-dependent manner in C6 cells. **c** σ-1R was expressed in C6 cells. **d** Pretreatment of C6 cells with the σ-1R antagonist (BD1047; 10 μM) inhibited methamphetamine-induced expression of p-Src. **e** Pretreatment of C6 cells with the σ-1R antagonist (BD1047; 10 μM) or the Src inhibitor (PP2; 10 μM) inhibited methamphetamine-induced expression of p-ERK. Representative immunoblots and the densitometric analysis of p-Src/t-Src from three separate experiments are presented. All the data are presented as the mean ± SD of three individual experiments. *p < 0.05 and **p < 0.01 compared with control group; #*p* < 0.05 compared with methamphetamine-treated group

Because methamphetamine increased the expression of HMGB1 in C6 cells and induces the activation of the Src and ERK pathway in primary mouse astrocytes [3], we next determined if the Src/ERK pathway regulates HMGB1 expression in C6 cells. Exposure of C6 cells to methamphetamine resulted in increased phosphorylation of Src and ERK with a peak response at 15 min (Fig. 2a, b). Our previous study indicated that σ-1R is expressed in primary astrocytes [3]. Consistent with this finding, C6 cells also expressed σ-1R (Fig. 2c). To investigate whether σ-1R is involved in methamphetamine-induced Src phosphorylation, C6 cells were pretreated with the σ-1R antagonist BD1047 followed by methamphetamine treatment. As shown in Fig. 2d, e, pretreatment of C6 cells with BD1047 (10 μM) significantly inhibited the phosphorylation of Src and ERK. Moreover, we also tested if Src activation is upstream of the ERK pathway. As shown in Fig. 2e, methamphetamine-induced phosphorylation of ERK was significantly inhibited by the Src inhibitor PP2 (10 μM). Consistent with our previous findings, methamphetamine also induced the activation of the Src/ERK MAPK pathway via σ-1R in C6 cells.

### Methamphetamine activates the NF-κB p65 transcription factor

**Fig. 3:**
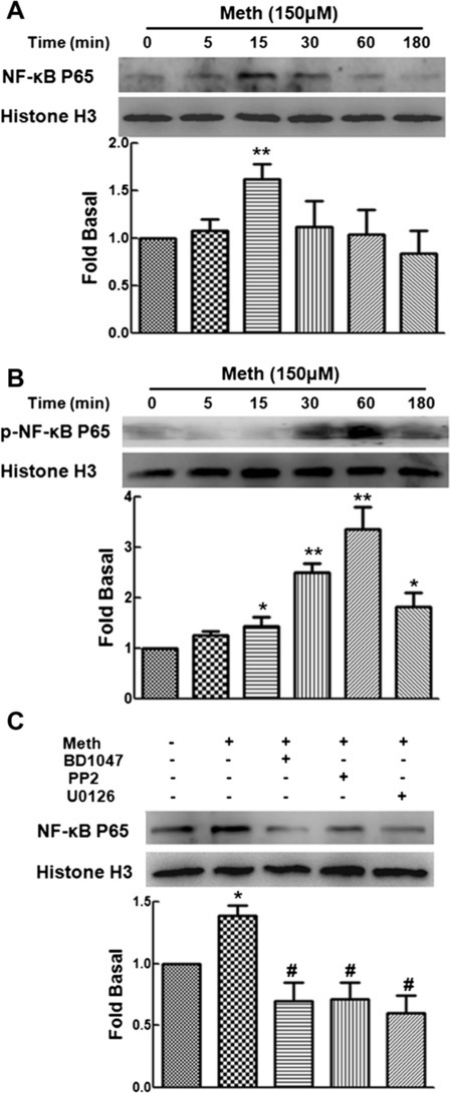
Methamphetamine induces NF-κB p65 transcription factor activation. **a** Effect of methamphetamine on translocation of NF-κB p65 into the nucleus in C6 cells. **b** Effect of methamphetamine on phosphorylation of NF-κB p65 in C6 cells. **c** Pretreatment of C6 cells with the σ-1R antagonist (BD1047; 10 μM), the Src inhibitor (PP2; 10 μM), or the ERK inhibitor (U0126; 10 μM) significantly inhibited methamphetamine-mediated translocation of NF-κB p65 into the nucleus. Representative immunoblots and the densitometric analysis of NF-κB p65/Histone H3 from three separate experiments are presented. All the data are presented as the mean ± SD of three individual experiments. *p < 0.05 and **p < 0.01 compared with control group; #*p* < 0.05 compared with methamphetamine-treated group

A previous study has indicated that NF-κB p65 activation is involved in HMGB1 expression [28]. Thus, we examined the effect of methamphetamine on the activation of NF-κB p65. As shown in Fig. 3a, methamphetamine treatment resulted in NF-κB p65 translocation into nucleus with a peak response at 15 min, since NF-κB p65 activity and nuclear translocation are regulated by their phosphorylation. Therefore, we further examine the effect of methamphetamine on the phosphorylation of NF-κB p65 in the nucleus of cells. As shown in Fig. 3b, treatment of primary human astrocytes with methamphetamine resulted in increased the phosphorylation of NF-κB p65 in the nucleus.

Since we found that methamphetamine induced the activation of the Src/ERK MAPK pathway via σ-1R, we next tested if these pathways are involved in NF-κB p65 translocation into the nucleus. As shown in Fig. 3c, the methamphetamine-induced translocation of NF-κB p65 into the nucleus was significantly inhibited by pretreatment with the σ-1R antagonist (BD1047; 10 μM), the Src inhibitor (PP2; 10 μM), and the ERK inhibitor (U0126; 10 μM). Taken together, these results suggested that methamphetamine-mediated NF-κB p65 activation lies downstream of the activation of the Src/ERK MAPK pathway though σ-1R.

### Src/ERK/NF-κB p65 pathway is involved in methamphetamine-induced HMGB1 expression

**Fig. 4:**
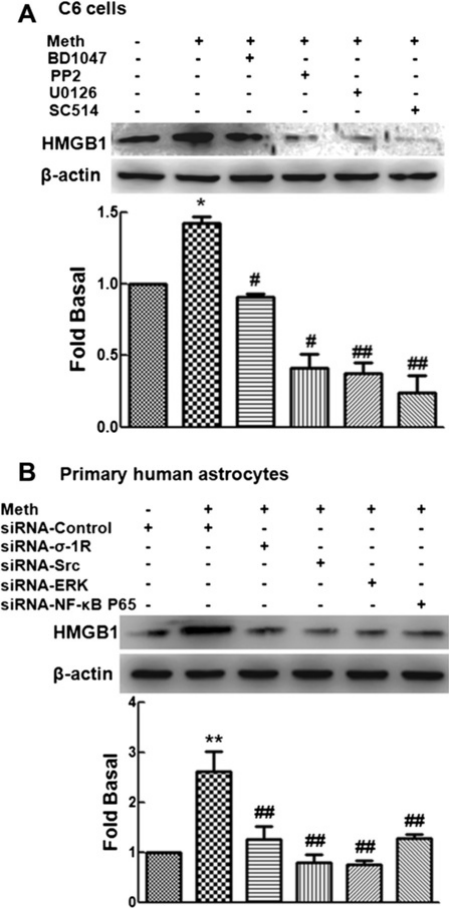
Src/ERK/NF-κB p65 pathway is involved in methamphetamine-induced HMGB1 expression. **a** Pretreatment of C6 cells with the σ-1R antagonist (BD1047; 10 μM), the Src inhibitor (PP2; 10 μM), the ERK inhibitor (U0126; 10 μM), or the Ikk-2 inhibitor (SC514; 10 μM) resulted in inhibition of the methamphetamine-mediated expression of HMGB1. **b** Methamphetamine-induced HMGB1 expression was attenuated by knockdown of σ-1R, Src, ERK, and NF-κB p65 in primary human astrocytes using specific siRNAs. Representative immunoblots and the densitometric analysis of HMGB1/β-actin from three separate experiments are presented. All the data are mean ± SD of three individual experiments. *p < 0.05 and **p < 0.01 compared with control group; #*p* < 0.05 and ##*p* < 0.01 compared with methamphetamine-treated group

Because methamphetamine up-regulated the expression of HMGB1 and activated the Src/ERK/NF-κB p65 pathway, we next investigated the link between HMGB1 expression and the Src/ERK/NF-κB p65 pathway. We pretreated C6 cells with the σ-1R antagonist (BD1047), the Src inhibitor (PP2), the ERK inhibitor (U0126), or the Ikk-2 inhibitor (SC514) for 1 h followed by treatment with methamphetamine for an additional 3 h. As shown in Fig. 4, the increased expression of HMGB1 mediated by methamphetamine was significantly inhibited by pretreatment with the σ-1R antagonist (BD1047; 10 μM), the Src inhibitor (PP2; 10 μM), the ERK inhibitor (U0126; 10 μM), or the Ikk-2 inhibitor (SC514; 10 μM) (Fig. 4a). Further validation of the involvement of these pathways in this process was confirmed by transfection of cells with siRNA σ-1R, Src, ERK, and NF-κB p65 followed by exposure to methamphetamine. As expected, methamphetamine-mediated induction of HMGB1 were attenuated by siRNA σ-1R, Src, ERK, and NF-κB p65 (Fig. 4b). Taken together, these findings thus underscore the involvement of σ-1R, Src, ERK, and NF-κB p65 cascade in methamphetamine-mediated induction of HMGB1 in astrocytes.

### Methamphetamine-induced HMGB1 mediates the activation of astrocytes

**Fig. 5:**
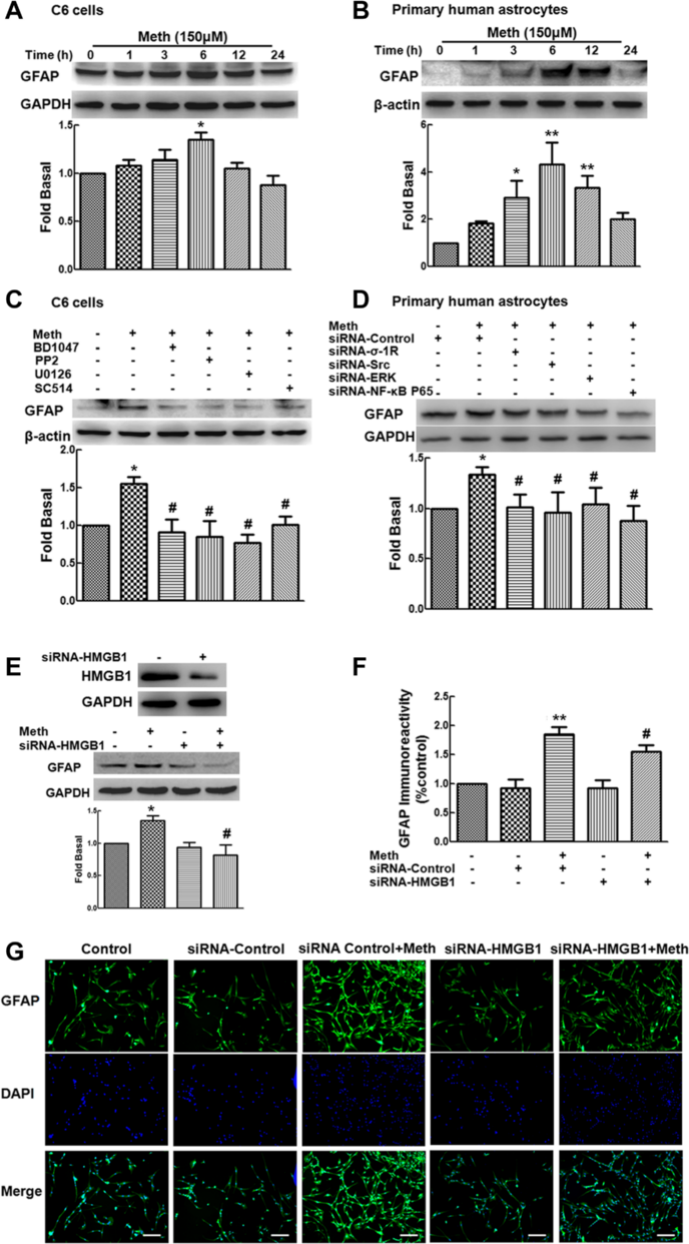
Methamphetamine-induced HMGB1 mediates activation of astrocytes. Methamphetamine (150 μM) increased the expression of GFAP in C6 cells (**a**) and primary human astrocytes (**b**). **c** Pretreatment of C6 cells with the σ-1R antagonist (BD1047; 10 μM), the Src inhibitor (PP2; 10 μM), the ERK inhibitor (U0126; 10 μM), or the Ikk-2 inhibitor (SC514; 10 μM) significantly reversed the increased GFAP expression induced by methamphetamine. **d** Transfection of primary human astrocytes with siRNA σ-1R, Src, ERK and NF-κB p65 resulted in attenuation of methamphetamine-induced GFAP expression. **e** Transfection of C6 cells with HMGB1 siRNA successfully decreased the expression of HMGB1 (*upper panel*). Knockdown of HMGB1 expression significantly inhibited the activation of C6 cells as determined by GFAP expression using western blot (*lower panel*). **f**–**g** Fluorescent intensity of GFAP was quantified in five areas using Image J software (**f**). Representative image of GFAP staining in C6 cells transfected with siRNA control or HMGB1 followed by treatment with or without methamphetamine (**g**). *Scale bars* all indicate 50 μm. All the data are mean ± SD of three individual experiments. *p < 0.05 and **p < 0.01 compared with control group; #*p* < 0.05 compared with methamphetamine-treated group in C6 cells or primary human astrocytes

HMGB1 was up-regulated in astrocytes treated with methamphetamine. Our previous study indicated that methamphetamine induces the activation of astrocytes [3]. Therefore, we next investigated the role of HMGB1 in the activation of astrocytes. Treatment of cells with methamphetamine induced astrocyte activation as indicated by the increased expression of GFAP with a peak response at 6 h in both C6 cells (Fig. 5a) and primary human astrocytes (Fig. 5b). Moreover, the increased expression of GFAP was significantly inhibited by the σ-1R antagonist (BD1047), the Src inhibitor (PP2), the ERK inhibitor (U0126), and the Ikk-2 inhibitor (SC514) (Fig. 5c). Meanwhile, increased GFAP expression induced by methamphetamine was also attenuated by siRNA σ-1R, Src, ERK, and NF-κB p65 in primary human astrocytes (Fig. 5d).

We next explored the role of HMGB1 in methamphetamine-induced activation of astrocytes. Transfection of C6 cells with HMGB1 siRNA successfully decreased the expression of HMGB1 as shown in Fig. 5e. Notably, knockdown of HMGB1 expression significantly reduced the activation of astrocytes as determined by the expression of GFAP assessed using western blot (Fig. 5e). This finding was further confirmed by immunostaining. As shown in Fig. 5f, g, methamphetamine treatment increased the expression of GFAP, which was attenuated by transfection with siRNA HMGB1. These findings clearly demonstrated that HMGB1 is involved in the activation of astrocytes induced by methamphetamine.

### HMGB1-mediated migration of astrocytes induced by methamphetamine

**Fig. 6:**
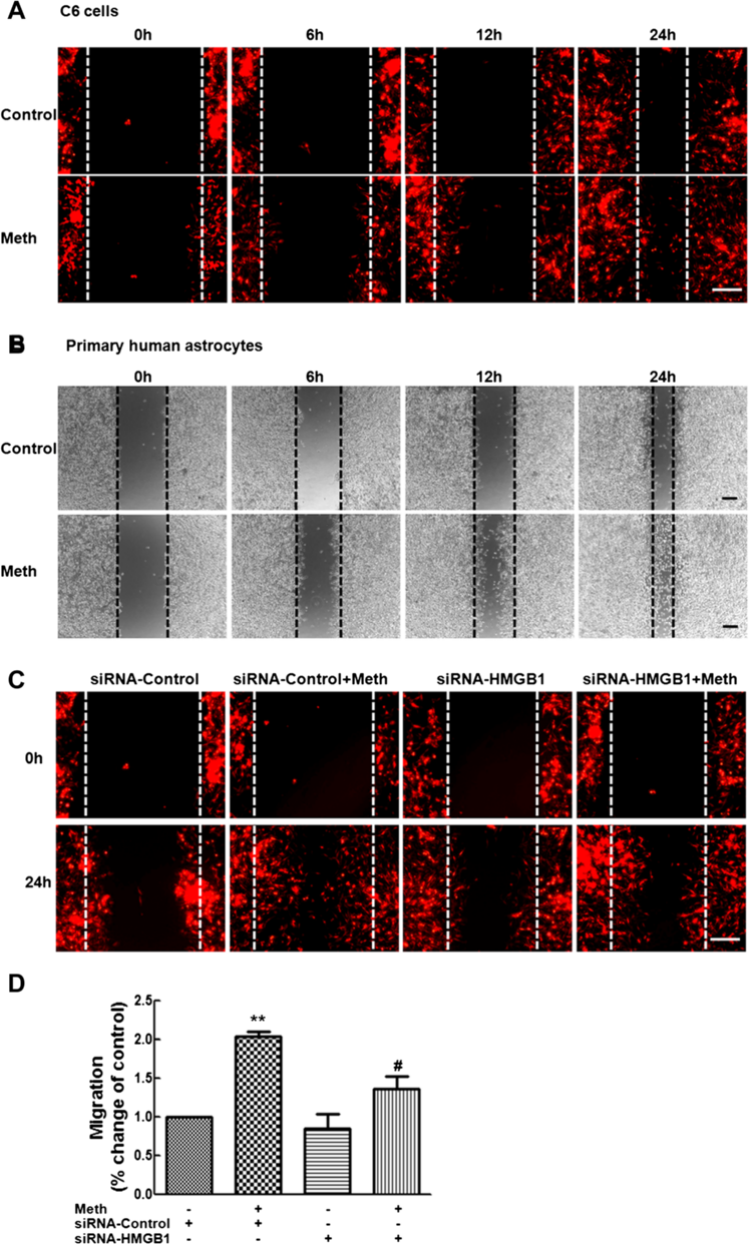
Methamphetamine induces HMGB1-mediated migration of astrocytes. The wound-healing assay showed that methamphetamine increased cell migration in a time-dependent manner in C6 cells (**a**) as well as in primary human astrocytes (**b**). **c**–**d** Transfection of C6 cells with siHMGB1 resulted in the inhibition of the methamphetamine-mediated induction of C6 cell migration. *Scale bars* all indicate 500 μm. The gap width from three separate experiments was quantitatively evaluated using Image J software. All the data are mean ± SD of three individual experiments. **p < 0.01 compared with control group; #*p* < 0.05 compared with methamphetamine-treated group in C6 cells

In addition to the activation of astrocytes, reactive astrocytes also migrate to the injured sites and orchestrate the inflammatory response. Therefore, we next determined the role of HMGB1 in the migration of astrocytes mediated by methamphetamine. A wound-healing assay showed that methamphetamine increased astrocyte migration in a time-dependent manner in C6 cells (Fig. 6a) as well as primary human astrocytes (Fig. 6b). Transfection of the cells with HMGB1 siRNA resulted in the inhibition of the methamphetamine-induced the migration of C6 cells (Fig. 6c, d), thereby supporting the role of HMGB1 in this process.

## Discussion

The present study demonstrated that (1) methamphetamine increases the expression of HMGB1 and that (2) HMGB1 promotes the activation and migration of C6 astrocytes. Up-regulation of HMGB1 in reactive astrocytes may contribute to the activation and migration of astrocytes through an autocrine feedforward mechanism that increases HMGB1 expression, thus amplifying the neuroinflammatory cascade induced by methamphetamine. Although previous studies have demonstrated that HMGB1 functions as a damage-associated molecular pattern (DAMP) involved in inflammatory response, it is still remained unclear whether HMGB1 is involved in methamphetamine-induced neuroinflammation.

In the current study, we demonstrated that methamphetamine exposure increased HMGB1 expression in astrocytes via the methamphetamine cognate receptor, σ-1R, which interacted with Src and activated the downstream MAPK/ERK pathway and the NF-κB p65 transcription factor leading to HMGB1 expression with subsequent functional activation and migration of astrocytes. To the best of our knowledge, these results demonstrated for the first time the critical role of HMGB1 in methamphetamine-mediated activation and migration of astrocytes. Thus, these findings imply that HMGB1 is a promising therapeutic target for amelioration of the methamphetamine-mediated neuroinflammation orchestrated by astrocytes.

Additionally, our study is the first to demonstrate that methamphetamine induced the expression of HMGB1 in astrocytes via σ-1R, as pretreatment of cells with the σ-1R antagonist BD1047 or knockdown of σ-1R abrogated increased expression of HMGB1 mediated by methamphetamine. Intriguingly, activation of σ-1R with methamphetamine subsequently resulted in phosphorylation of the Src tyrosine kinase. Our previous study indicated that methamphetamine mediates the activation of Src through activation of σ-1R [3]. Inhibition of Src activation with the Src inhibitor-PP2 as well as siRNA Src significantly blocked the methamphetamine-mediated increased expression of HMGB1, thereby suggesting that Src activation was upstream of the methamphetamine-induced HMGB1 expression. To our knowledge, this is the first evidence that Src activation is involved in the regulation of HMGB1 expression, which contradicts a previous study indicating that Src activation lies downstream of HMGB1 [29].

We also examined the signaling pathways involved in methamphetamine-mediated up-regulation of HMGB1. Our previous studies indicated that methamphetamine induces ERK phosphorylation [30–32]. Using both pharmacological and genetic approaches, our findings demonstrated that the ERK pathway is involved in the methamphetamine-mediated increased expression of HMGB1 (Fig. 4), which was consistent with a previous study indicating that ERK activation is involved in the expression of HMGB1 induced by IL-1β in primary cortical astrocytes [33]. However, Ding et al. demonstrated that p38 MAPK, but not the ERK pathway, is involved in the expression of HMGB1 in lung injury induced by LPS [34]. The different findings between Ding et al. and our current study may be due to the different cell systems examined.

NF-κB p65 is a dimeric protein widespread in the cytoplasm. Through gene products and downstream signaling pathways, NF-κB p65 participates in numerous pathological processes, such as inflammation, immune response, apoptosis, cell differentiation, tumor regulation, and cell cycle regulation [35–37]. Our previous study indicated that methamphetamine induces activation of NF-κB p65, a transcription factor that is downstream of ERK activation [3]. Consistent with these findings, our current study also indicated that methamphetamine exposure induces translocation of NF-κB p65 into the nucleus via σ-1R with subsequent activation of Src and the MAPK/ERK cascade in rat C6 cells. Interestingly, blockade of NF-κB p65 signaling significantly inhibited the methamphetamine-mediated up-regulation of HMGB1 (Fig. 5). This finding was consistent with the previous study demonstrating that NF-κB p65 is involved in HMGB1 expression [28]. However, increasing evidence has indicated that NF-κB p65 lies downstream of HMGB1 resulting in enhancement of migration via the activation of MMP-9 [38].

It is well-recognized that astrocyte activation and migration are key features of reactive astrogliosis. Cell migration and morphological changes are closely associated with chronic activation of astrocytes. Activated astrocytes often show characteristic changes in migratory and morphological phenotypes, which are collectively referred to as reactive astrogliosis. Molecular mechanisms underlying reactive astrogliosis have been the subject of intensive investigation [39]. The reaction of astrocytes is characterized by early proliferation and increased expression of GFAP. A previous study has reported that HMGB1 promote the proliferation and migration of glioma cells [22]. Moreover, HMGB1 is involved in pulmonary artery remodeling by enhancing proliferation and migration of smooth muscle cells [40]. Regarding the detailed mechanisms underlying the migration induced by HMGB1, Nehil et al. reported that HMGB1 promotes tumor cell migration through epigenetic silencing of semaphorin 3A [41]. Our studies provide evidence for methamphetamine-mediated activation of astrocytes with concomitant increased expression of HMGB1 in astrocytes (Fig. 5). The significance of this study is that it is the first to provide evidence using genetic approaches that HMGB1 plays a key role in methamphetamine-mediated astrocyte activation and migration. Our study indicated that HMGB1 is involved in methamphetamine-mediated activation and migration of astrocytes. However, in contrast to our finding, Zuo et al. reported that HMGB1 inhibits cell motility and metastasis by suppressing activation of the transcription factor, CREB, and subsequent nWASP expression [42]. A possible explanation for the different function of HMGB1 may be attributed to the different cell types in these studies.

HMGB1 and its receptor, receptor for advanced glycation end products (RAGE), are pivotal factors in the development and progression of many types of tumors [43]. A previous study has indicated that HMGB1 triggers neuronal death by directly activating RAGE signaling cascades [44]. RAGE is a multi-ligand receptor and is involved in various physiological processes, such as inflammation and development [29]. RAGE is expressed in neurons, glia, and endothelial cells. However, it needs to be further investigated if RAGE signaling is involved in HMGB1-mediated astrocytic cell activation and migration. Moreover, although the present study provided the detailed underlying mechanisms by which methamphetamine increases the expression of HMGB1, the precise mechanism by which HMGB1 promotes activation and migration needs to be elucidated.

## Conclusions

**Fig. 7:**
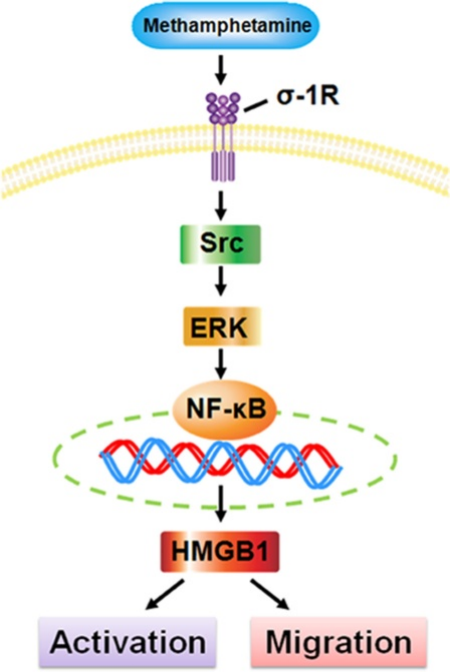
Schematic of signaling pathways involved in methamphetamine-induced astrocyte activation via an autocrine mechanism of HMGB1 expression. Exposure of astrocytes to methamphetamine leads to activation and migration of astrocytes via up-regulation of HMGB1. Inhibition of σ-1R, Src, or ERK MAPKs results in the subsequent inactivation of the downstream NF-κB p65 transcription factor. NF-κB p65 enhances HMGB1 expression and subsequently induces astrocyte activation and migration, thereby amplifying the methamphetamine response

In summary, our findings outlined the detailed molecular pathway involved in methamphetamine-mediated activation and migration of astrocytes via σ-1R with downstream activation of Src and MAPK/ERK pathways and subsequent activation of NF-κB p65 resulting in increased HMGB1 expression (Fig. 7). These findings have implications for activated astrocytes induced by methamphetamine. Targeting HMGB1 can be considered as a therapeutic strategy for treatment of methamphetamine-mediated neuroinflammation.

## Abbreviations

CNS: central nervous system
DMEM: Dulbecco’s modified Eagle’s medium
FBS: fetal bovine serum
GFAP: glial fibrillary acidic protein
HMGB1: high-mobility group box 1
LV-RFP: lentivirus containing red fluorescent protein
NGS: normal goat serum
OD: optical density
PBS: phosphate-buffered saline
PVDF: polyvinylidene difluoride
σ-1R: sigma-1 receptor
